# Problematic Attributions of Entropic and Hydrophobic Effects in Drug Interactions

**DOI:** 10.1021/acsbiomedchemau.4c00148

**Published:** 2025-04-11

**Authors:** Hans-Jörg Schneider

**Affiliations:** 9379 FR Organische Chemie der Universität des Saarlandes, D 66123 Saarbrücken, Germany

**Keywords:** drug binding, noncovalent interactions, entropic and hydrophobic effects, dispersive van der Waals contributions, supramolecular complexes, drug finding, artificial intelligence

## Abstract

The ΔG affinity of drugs with biopolymers and the underling noncovalent interactions play an essential role in drug discovery. Supramolecular complexes can be designed for the identification and quantification of specific interactions, including their dependence on the medium; they also secure the additivity of ΔΔG increments. Such analyses have helped to clarify hydrophobic effects in intermolecular associations, which are barely measurable with small alkyl groups, but large in the presence of curved surfaces in which the replacement of hydrogen bond-deficient water molecules by a ligand leads to sizable enthalpy gain. Difficult to predict entropy contributions TΔS to ΔG vary between 5% and over 90%, particularly in drug associations, as is obvious from literature data. As illustrated with several drug complexes, many so-called hydrophobic effects involve in fact van der Waals or dispersive interactions. Measurements with supramolecular porphyrin complexes allowed us to derive dispersive binding contributions for many groups, which exhibit a correlation with polarizability. In consequence, heteroatoms or π-systems always lead to enhanced van der Waals contributions, while for hydrophobic effects the opposite is expected. Binding contributions from supramolecular complexes can in the future also help artificial intelligence approaches in drug discovery, by expansion of hybrid databases with potential ligands containing groups with desired binding contributions.

## Graphical abstract

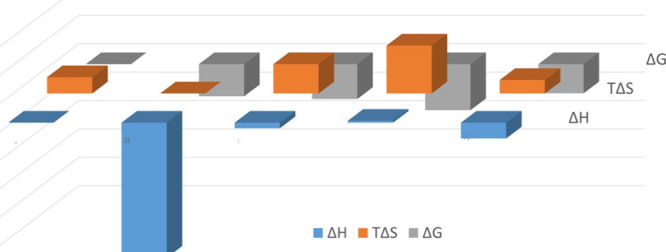

## Introduction

The discussion of noncovalent interactions relating to drug design and drug finding suffers from frequent neglect or conflicting interpretations of entropic and hydrophobic effects. Both contributions play, besides ion pairing, a major role in aqueous surroundings and are addressed in the present article with an emphasis on their possible binding mechanisms. For the efficiency of drugs, their binding strength to bioreceptors plays an important role and is usually defined as free interaction energy ΔG, also in multivalent systems where several binding sites enhance affinities.^1–6^ Other important factors such as drug solubility and drug permeation through barriers were efficiently quantified by, for example, H-bond acidity and H-bond basicity values derived by Abraham, Raevsky and others from distribution or gas–liquid partition coefficients.^7–10^ Hydrogen bonding is weak in protic media, for which reason we discuss here only entropic, hydrophobic, and van der Waals or dispersive effects, which, besides ion pairing, dominate interactions in water.

In recent decades, the empirical evaluation of underlying noncovalent interactions has made considerable progress on the basis of supramolecular complexes (see Table S1), in which the nature and number *n* of specific interactions can be systematically varied, also in different media.^11,12^ As illustrated in the Supporting Information (Figures S1–S5) one usually observes linear correlation between the measured total ΔG values and the number *n,* which allows one to derive a single value for the specific interaction energy, and also secures additivity of single ΔΔG values.^13^ The same strategy has been established for a long time by Hammett^14^ with linear free energy correlations, where reactivity values are derived from substituent effects. As with Hammett-type relations and observed also in the analysis of intramolecular noncovalent interactions,^15^ additive ΔΔG values are similar and additive in different model systems, while those of TΔS and enthalpic ΔH contributions are not. Affinity ΔG values for the most important noncovalent interactions are available from many equilibrium measurements of supramolecular complexes;^16^ such ΔG values may be used in the design of new drugs and the underlying interaction mechanisms.

## Entropy Effects

A major and until now largely unresolved problem is the contribution of entropic factors TΔS to the total affinity ΔG,^3^ for which measurements show 5% to 90% contribution to the total ΔG value with supramolecular complexes,^17^ including ionophores^18^ or cyclodextrin.^19^ Drug interactions with biopolymers^20–24^ show an even larger variety,^22,25,23^ as exemplified by extensive measurements (Table 1).^22^ Computational approaches of entropic contributions to ligand binding to proteins must take into account hydration effects^20,21,26,27^ as well as fluctuation of the interaction energy and the flexibility of biopolymers as essential factors, but still show large differences to experimental TΔS and ΔH values.^28^ Noticeably, the ΔH and TΔS contributions for drug binding to biopolymers were found to be significantly temperature dependent.^22^ Isothermal titration calorimetry (ITC) has become a promising yet still not very frequently used tool for the analysis of drug binding.^29,22^ As shown in Table 1,^22^ drug interactions with biopolymers can be either enthalpy or entropically driven, while supramolecular complexes exhibit mostly adverse TΔS contributions, with the exception of salt bridges. Fairly linear correlations between ΔH and TΔS are observed if the interaction mechanisms are similar and/or if the receptor nature is similar, like with cyclodextrins. Several explanations for the ΔH – TΔS compensation have been put forward.^30^ Intuitively a strong binding force, ΔH is expected to lead to less flexibility or to smaller degrees of freedom, but a general occurrence of such compensations has also been declared as phantom.^31^

**Table 1:** Selected Thermodynamic Parameters for Drug Interactions with Biopolymers Measured by Isothermal Titration Calorimetry (ITC)^22^ ^a^

	T (K)	ΔH (kJ mol^–1^)	TΔS (kJ mol^–1^)	ΔG (kJ mol^–1^)
(1) NOF with HSA	288	–15.0	11.4	–26.4
(2) 3HF with HSA	293	–95.1	–72.5	–22.6
(3) NHM with DNA	288	–3.9	20.6	–24.5
(4) NHM with RNA	293	1.3	33.7	–32.4
(5) CRYP with RNA	288	–11.1	9.45	–20.5

a (1) Indoloquinoline alkaloid (NOF) with human serum albumin; (2) 3-hydroxyflavone 3HF with human serum albumin; (3)­β-carboline drug norharmane ( 9*H*-Pyrido­[3,4-*b*]­indol) NHM with DNA; (4) β-carboline drug norharmane NHM with RNA; (5) indoloquinoline alkaloid cyrptolepine (CRYP) with RNA.

The interaction of *N*-phenylpiperazine derived drugs with an alpha-1-adrenoceptor protein presents a case of an unusually large positive entropy contributions as the driving force, with small, even endothermic enthalpy values.^32^ It is also an example of the application of ΔΔG free energy increments derived from supramolecular complexes for an alternative prediction of the total affinity. The observation of Δ*H* ^*θ*^ < 0 and Δ*S* ^*θ*^ > 0 was attributed to electrostatic forces being the main factor for binding.^32^ However, the pyrimidine ring with amidine functions in the drugs present strong bases; in combination with the piperazine moiety protonation provides for ligands of type A (Scheme 1) with three cationic centers, allowing for examples with aspartic acid Asp of the protein 3 × 2 = 6 salt bridges with a value of ΔΔG = 5 ± 1 kJ/mol (Figures S1, S2, S3). In water at moderate ionic strength, one then expects an affinity value of ΔG = 6 × 5 = 30 kJ/mol,^33^ quite close to the reported experimental free energy of 27 to 30 kJ/mol for derivatives 1 to 6; ligands of type B lack the pyrimidine unit and exhibit as expected with ΔG = 24 kJ/mol, a correspondingly lower affinity. Clearly, the thermodynamic values indicate ion pairing or salt bridges as major binding factors, in contrast to complexation due to electrostatic forces; these, like those based on hydrogen bonds or donor–acceptor interactions, are well-known to be driven by enthalpy, usually against adverse entropic factors.

**Scheme 1:**
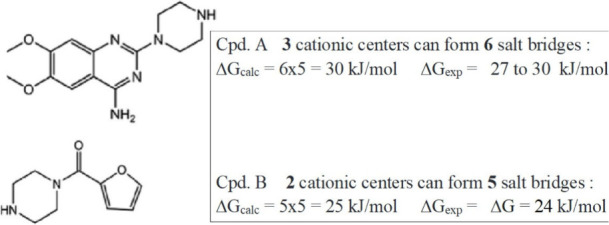
N-Phenylpiperazine Derived Drugs,^32^ Salt Bridges with Aspartic Acid Residues of an Alpha-1-Adrenoceptor Protein

## The Dichotomy of Hydrophobic and Dispersive Contributions

In relation to drug interactions, hydrophobic effects^22,34–37^ are quoted more often than van der Waals or dispersive interactions,^38–54^ of which many also concern drug delivery and pharmacokinetics. It has been stated that while dispersion interactions are well understood in model systems, it is not yet possible to test calculations for protein interactions.^55^ Molecular dynamics simulations of peptides omitting hydrogen bonding in the peptide-solvent system indicate that folding into helical and hairpin-like structures is also possible on the basis of dispersive interactions.^56^ In the past years, special secondary interactions were found to influence protein folding.^57^ These include C–H···O hydrogen bonding, n→π* interactions, halogen and chalcogen bonding, and interactions involving arenes which actually are also of dispersive nature.

Both hydrophobic and dispersion interactions can play an important role in aqueous media; due to the low polarizability of water molecules, they are most often summarized as hydrophobic effects, although their distinction is by no means a matter of semantics. In fact, they refer to an opposite interaction mechanism, as is visible in the very different water solubility of, for example, alkanes and oligopeptides of similar size; due to the presence of more polarizable amide functions, the latter can in contrast to alkanes well undergo dispersive interactions.

Several approaches for the characterization of hydrophobic interactions and corresponding scales of hydrophobicity are used in many biomedical publications.^58^ Wolfenden et al. developed an early scale on the basis of partitioning small molecule analogs of amino acid side chains between water and cyclohexane.^59,60^ White developed a peptide-based system for interactions between side chains and lipids,^61^ using traditional water and 1-octanol partition coefficients.^62^ Other methods use interactions with lipophilic membranes as the hydrophobicity model.^63^ The binding strength of drugs with serum albumin was found to correlate with the drug hydrophobicity,^64^ and was attributed to the drug’s ability to desolvate the protein binding site.^65^

## Distinction of Hydrophobic and Dispersive Interactions with Supramolecular Model Complexes: Nonclassical Hydrophobic Effects

Measurements of equilibria in supramolecular complexes with water-soluble porphyrins as receptors have allowed researchers to clearly distinguish hydrophobic and dispersive interactions.^66^ Alkanes such as propyl residues show negligible affinities to the hydrophobic porphyrin surfaces, cyclohexyl residues contribute less than ΔG = 1 kJ/mol, but a single phenyl group exhibits an affinity of ΔG = 8 kJ/mol; a similar value is reached, for example, with small peptides such as pentaglycine.^17^ The almost negligible affinity of alkanes allows one to quantify the dispersive contributions of different functions by hundreds of equilibrium data points, which as expected exhibit a roughly linear correlation with corresponding polarizability values.

The absence of hydrophobic interactions from alkyl residues with the flat porphyrin surface shows that such attractive hydrophobic forces do not exist but do not generally eliminate hydrophobic effects. These can be large if the hydrophobic surface is not flat but curved; in the corresponding cavities of the surface, there are water molecules with less than the optimal number of hydrogen bonds. The replacement of such frustrated high energy water molecules by a suitable guest molecule can lead to large affinities,^67,68^ characterized by a significant enthalpy gain (see Figure 1).^21,69,70^ Such enthalpy driven hydrophobic associations with a decrease of heat capacity are usually characterized as nonclassical effects, contrasting the classical model of hydrophobic effects driven by entropic contribution changes and negative changes of heat capacity.^71–75^ Classical effects are seen in the exothermic but entropically unfavorable dissolution of simple gases and hydrocarbons in water, while nonclassical effects are characterized by endothermic hydration of nonpolar pockets and cavities which contain hydrogen-bond deficient or high-energy water.^76–78^

**Figure 1:**
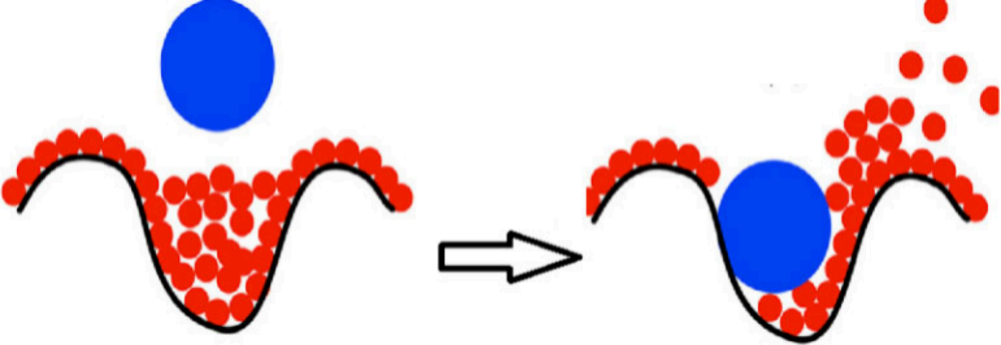
Illustration of a nonclassical hydrophobic effect by replacement of hydrogen-bond deficient high-energy water molecules by a ligand. Adapted with permission from ref 21. Copyright 2019 American Chemical Society.

A problem for the computation of dispersive effects is that polarizability numbers for functions such as amides are not simply additive for participating atoms,^79^ while experimental data from measurements with porphyrins as the model show sizable values for amides which influence peptide and protein interactions. Suitable increments were deduced for force fields for amide functions with applications for peptides and proteins;^80,81^ the advanced MM4 force field was successfully tested by comparison with gas phase or crystal structures, and with vibrational spectra and by crystal sublimation heats.^82^ The AMBER force field was used in combination with MD simulations also for van der Waals interactions in proteins on the basis of experimental vaporization enthalpies and liquid densities of small molecules containing corresponding moieties in proteins.^83^

## Examples of Drug Complexations with Either Hydrophobic or Dispersion Contributions

The majority of publications on noncovalent interactions with drugs and protein attribute those to hydrophobic effects and less frequently to van der Waals or dispersive interactions as decisive. In drug resistant mutations on HIV-1 proteases, van der Waals interactions were ascribed also to interactions between alkyl residues of amino acids.^84^ The binding of primary alcohols to the major urinary protein is characterized by an enthalpy increase with increasing alcohol chain length and decreasing TΔS contribution which together with the linear dependence of both parameters was attributed to favorable dispersive protein–ligand interactions.^85^

The binding of the hydrophobic drug ibuprofen to lysocyme as a protein model is an example where interactions with lysozyme amino acids such as TRP, ILE and ALA were ascribed to dominating hydrophobic effects (Figure 2), with a total of 13 hydrophobic interactions with TRP62 (three), TRP63 (four), TRP108 (two), ILE98 (two), single binding with ILE58 and ALA107, and small hydrogen bonding through ASN59.^86^ However, dispersive contributions from the stacking between at least TRP61 and TRP62 with the iboprofen arene are expected to yield already about ΔG = 2 × 8 = ca. 16 kJ/mol, and hydrogen bonds with ASN59 and GLU53 can contribute up to at least 10 kJ/mol, yielding a ΔG value of up to at least 25 kJ/mol, not far from the experimental value. Noticeably, the binding of ibuprofen increased with increasing temperature. This would agree not only with hydrophobic effects but also with the dispersive nature of the interactions.

**Figure 2:**
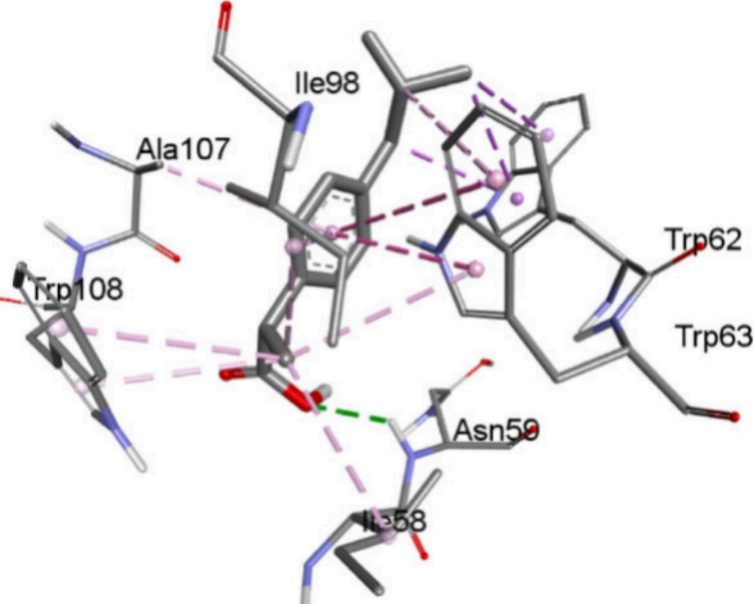
Docking of ibuprofen to lysocyme as protein model, with interactions ascribed to hydrophobic forces; ΔG = 23.4 kJ/mol. Adapted with permission from ref 86. Copyright 2023 Elsevier.

The binding of the more hydrophilic paracetamol *decreased* with increasing temperature, which agrees with more contributions from hydrogen bonds found for this compound. The binding energies were calculated by docking on the basis of accessible surface area of interacting residues and agreed well with experimental data, which seems to speak for prevailing hydrophobic interactions. However, the size and polarizability of phenyl residues resembles that of, for example, cyclohexyl moieties, which would mean that calculations based on dispersive interaction could lead to a similar interaction.

An example of mainly dispersive interaction was described for a protein synthase and an aminothiazole inhibitor, which occurs with a binding affinity of 25 μM, exhibiting not only dispersive S···aryl interaction with methionine (Met138), but also stacking between the inhibitor phenyl ring with the backbone amide groups of Ala162 and Cys163.^87^ Figure 3 illustrates the sulfur and amide group interactions with aromatic moieties.

**Figure 3:**
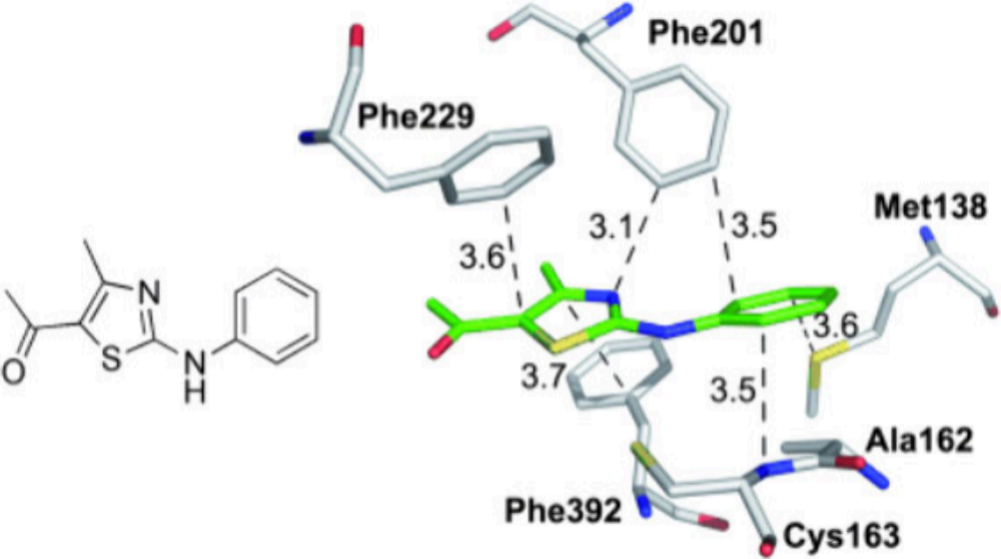
Dominating dispersive interactions of an aminothiazole inhibitor in a protein; typical distances in Å (based on X-ray structure, resolution 1.35 Å, PDB code: 2VBA). Adapted with permission from ref 87. Copyright 2011 Wiley VCH.

A case where van der Waals besides cation-π interactions were taken into account without any consideration of hydrophobic effects is the investigation of ethylpiperidinium iodides as antagonists for nicotinic acetylcholine receptor proteins (Figure 4).^88^ The antagonist in Figure 3 exhibits a large affinity with 36 kJ/mol in comparison to choline (16 kJ/mol) which was ascribed to cation-π interactions with the arenes of the amino acids tyrosine Y91, Y187, Y194, and tryptophan W147, W58, and to van der Waals interactions with leucine L106 and glutamine Q115. The interaction between the aliphatic chains of leucine L106 and of the antagonist speaks rather for hydrophobic effects, also in view of the observed dependence on the alkyl chain length; the interaction with glutamine Q115 is expected to be due to dispersive forces in view of the relatively large contributions of amide functions.^66^

**Figure 4:**
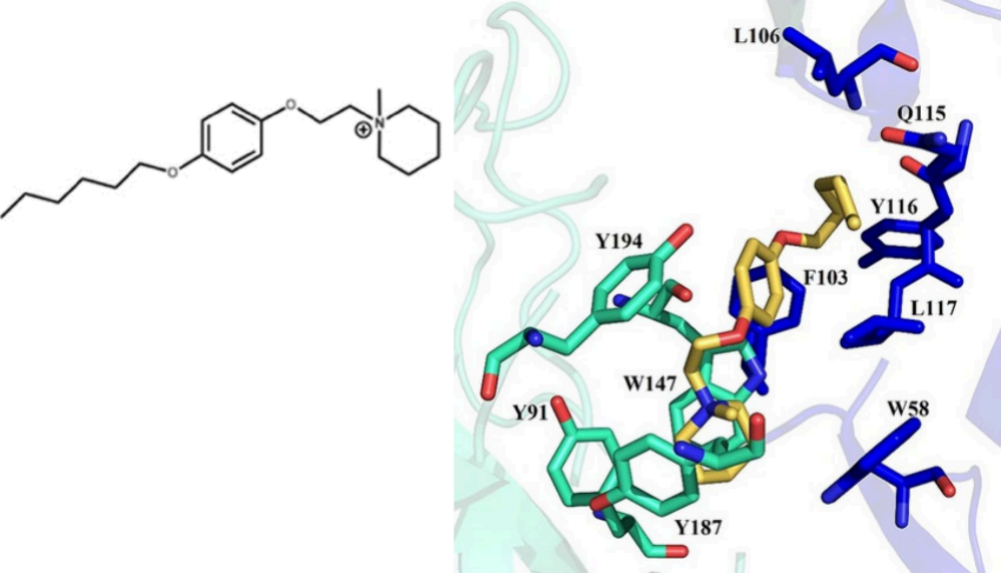
Interactions of a methylpiperidiniumiodide antagonist (yellow color) with the nicotinic acetylcholine receptor protein; see text for explanations see. Adapted from ref 88. Copyright 2018 Frontiers under the terms of the Creative Commons Attribution License (CC BY) .

The binding of different phenylacetamides to a serine protease factor was convincingly assigned to predominating dispersive contributions, particularly in view of the affinity increase with halogen substituents at the phenyl *p*-position; with R = Br and R = Cl, the inhibition showed a gain of 10.5 kJ/mol in comparison to R = H, but by only 3.5 kJ/mol for R = F. QM computations at the MP2 level and searches in crystal databases showed the absence of directional orientation, also indicating dominating dispersive contributions.

The folding of peptides was introduced as a model for proteins, and provides more direct access to the responsible contributions in protein folding.^89,90^ A particular peptide^43^ contains only amino acids with lipophilic side groups, which obviously speaks for hydrophobic and not dispersion interactions. The driving force for the folding is not necessarily an attraction between the amino acid alkyl groups but can also be due to the presence of high energy water molecules on the open structure of the peptide.

Unfolding of a-hairpin peptide bearing halogen substituents at a phenylalanine (Figure 5) exhibits an increasing stabilization of the hairpin, in the order F (0. 5) < Cl (1.42) < Br (1.97) < I (2.26, all numbers ΔΔG values in kJ/mol),^91^ very clear evidence for dispersive interactions.

**Figure 5:**
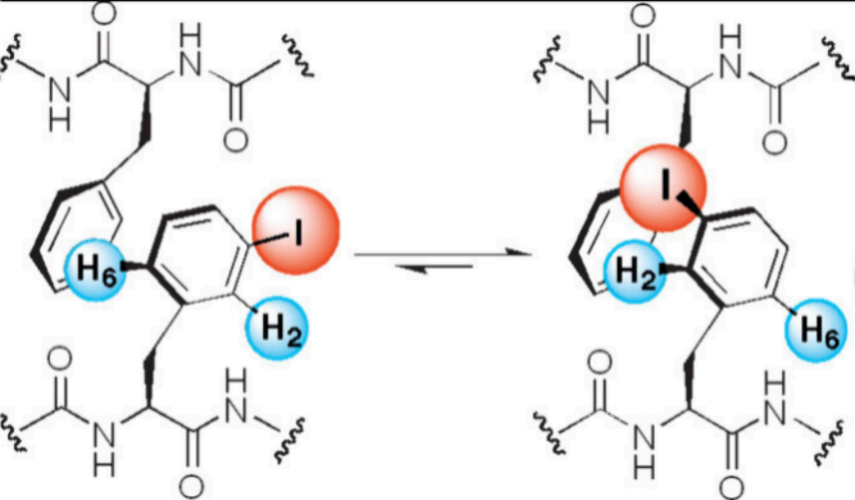
Peptide hairpin stabilization by dispersive iodine–aryl interaction. Adapted with permission from ref 91. Copyright 2004 American Chemical Society.

## Conclusions

Entropic contributions of TΔS to intermolecular binding affinity ΔG values can reach up 90% and are temperature dependent; entropy driven complexations accompanied by very small or even adverse enthalpic contributions indicate dominating ion pairing. Noncovalent interactions of drugs are often attributed to hydrophobic effects, while dispersive or van der Waals interactions are often overlooked. Classical hydrophobic contributions are characterized by dominating entropic values; enthalpic contributions may indicate nonclassical hydrophobic factors due to the presence of high energy water in the receptor. However, most drugs and all biopolymers contain always elements, at least heteroatoms or π-bonds, which decrease hydrophobicity and lead to sizable polarizability and therefore often to dispersive instead hydrophobic interactions. Nucleic acids offer, with nucleobases and heteroatoms in the bridges particularly, many functions, making them ideal candidates for dispersive interactions. Peptides and proteins invariably contain many amide functions; a single amide group alone exhibits a dispersive energy increment which is close to that of iodine or sulfur groups; side groups containing arenes, sulfur, or oxygen elements furthermore increase dispersive contributions. Oliogopeptides exhibit dispersive interactions with an affinity that corresponds simply to the sum of amide functions.

Most drugs contain elements that also will lend themselves to dispersive interactions, for which increments of binding affinity can be derived from measurements with porphyrin models. Classical hydrophobic effects are barely measurable between small alkane residues and flat receptor surfaces, such as arenes. Nonclassical hydrophobic effects are until now mostly attributed to enthalpy driven associations; they can materialize in the presence of high energy water molecules at a receptor surface which lacks the optimal number of hydrogen bonds.

Artificial Intelligence plays an increasing role in drug discovery;^92,93^ hybrid databases can serve for de novo drug design^94^ and can evaluate also drug affinity values.^95^ A hybrid AI approach uses a significantly enlarged hypothetic database by inclusion of a multitude of unknown structures containing groups with empirically predictable noncovalent interactions. Suitable groups can be selected from experiments with supramolecular complexes^1,6,17^ (Figures S1–S5) and from tabulations of, e.g., hydrogen bond associations.^7–9^

## Supplementary Material



## Notes

Ion pair association free energies in water; solvent effects on ion pairing/salt bridges; ion pair stability in water as a function of ionic strength, Debye-Hückel-correlation; ion pair distance dependence of binding free energy Δ*G*, measured in water, average 5 kJ/mol per salt bridge, linear dependence of the association energy Δ*G* (kJ/mol) of Et_4_NBr on dielectric constant with 1/e; hydrogen bond ΔΔG increments reflect electrostatic interactions, correlation between hydrogen bond increments ED and free energies ΔG of crown and cryptand complexes; temperature dependence of relevant thermodynamic parameters; typical binding free energies for important noncovalent interactions from supramolecular complexes (PDF)

## References

[1] Multivalency: Concepts, Research & Applications; Huskens, J. ; Prins, L. ; Haag, R. ; Ravoo, B.J. , Eds.; John Wiley & Sons Ltd., 2018.

[2] Mammen M., Choi S.-K., Whitesides G. M. (1998). Polyvalent
Interactions in Biological
Systems: Implications for Design and Use of Multivalent Ligands and
Inhibitors. Angew. Chem., Int. Ed..

[3] Badjic J. D., Nelson A., Cantrill S. J., Turnbull W. B., Stoddart J. F. (2005). Multivalency
and cooperativity in supramolecular chemistry. Acc. Chem. Res..

[4] Mahon E., Barboiu M. (2015). Synthetic multivalency for biological applications. Org. Biomol Chem..

[5] Fasting C., Schalley C. A., Weber M., Seitz O., Hecht S., Koksch B., Dernedde J., Graf C., Knapp E.-W., Haag R. (2012). Multivalency as a chemical
organization and action principle. Angew. Chem.
Int. Ed. Engl..

[6] Schneider, H.-J. Additivity of Energy Contributions in Multivalent Complexes. In Multivalency: Concepts, Research & Applications; Huskens, J. ; Prins, L. ; Haag, R. ; Ravoo, B.J. , Eds.; John Wiley & Sons Ltd., 2018; p 1–21.

[7] Abraham M. H., Ibrahim A., Zissimos A. M., Zhao J., Comer Y. H., Reynolds D. P. (2002). Application of hydrogen
bonding calculations in property
based drug design *Drug Discov*. Today..

[8] Raevsky O., Skvortsov V. (2005). Quantifying hydrogen bonding in QSAR and molecular
modeling *SAR QSAR*. Environ.
Res..

[9] Laurence C., Berthelot M. (2000). Observations
on the strength of hydrogen bonding *Perspect*. Drug Discovery Des..

[10] Solomonov B. N., Yagofarov M. I. (2024). Can the hydrogen bonding enthalpy be calculated from
the binding constant at 298.15 K. J. Mol. Liq..

[11] Supramolecular Systems in Biomedical Fields; Schneider, H.-J. , Ed.; The Royal Society of Chemistry: Cambridge UK, 2013.

[12] Escobar L., Ballester P. (2021). Molecular
Recognition in Water Using Macrocyclic Synthetic Receptors. Chem. Rev..

[13] Schneider H.-J. (1994). Linear
Free Energy Relations and Pairwise Interactions in Supramolecular
Chemistry. Chem. Soc. Rev..

[14] Richard J. P., Cristobal J. R., Amyes T. L. (2021). Linear Free Energy Relationships
for Enzymatic Reactions:
Fresh Insight from a Venerable Probe. Acc. Chem.
Res..

[15] Elmi A., Cockroft S. L. (2021). Quantifying Interactions and Solvent Effects using
Molecular Balances and Model Complexes. Acc.
Chem. Res..

[16] Biedermann F., Schneider H.-J (2016). Experimental Binding Energies in Supramolecular Complexes. Chem. Rev..

[17] Schneider H. J. (2024). Distinction
and quantification of noncovalent dispersive and hydrophobic effects. Molecules.

[18] Arnaud-Neu F., Delgado R., Chaves S. (2003). Critical evaluation
of stability
constants and thermodynamic functions of metal complexes of crown
ethers - (IUPAC Technical Report). Pure Appl.
Chem..

[19] Rekharsky M. V., Inoue Y. (1998). Complexation thermodynamics of cyclodextrins. Chem. Rev..

[20] Breiten B., Lockett M. R., Sherman W., Fujita S., Al-Sayah M., Lange H., Bowers C. M., Heroux A., Krilov G., Whitesides G. M. (2013). Water Networks
Contribute to Enthalpy/Entropy Compensation
in Protein-Ligand Binding. J. Am. Chem. Soc..

[21] Al-Husseini J. K., Stanton N. J., Selassie C. R., Johal M. S. (2019). The binding of drug
molecules to serum albumin: the effect of drug hydrophobicity on binding
strength and protein desolvation. Langmuir.

[22] Paul B. K. (2022). Classical
vs. nonclassical hydrophobic interactions underlying various interaction
processes: Application of isothermal titration calorimetry *Chem*. Physics Impact.

[23] Meloun M., Ferencíková Z. (2012). Enthalpy–entropy compensation
for some drugs dissociation in aqueous solutions. Fluid Phase Eq..

[24] Gilli P., Ferretti V., Gilli G., Borea P. A. (1994). Enthalpy-entropy
compensation in drug-receptor binding. J. Physic.
Chemistry.

[25] Ladbury J. E., Klebe G., Freire E. (2010). Adding calorimetric data to decision
making in lead discovery: a hot tip *Nat*. Rev. Drug Discovery.

[26] Chandler D. (2005). Interfaces
and the driving force of hydrophobic assembly. Nature.

[27] Bischofberger I., Calzolari D. C. E., De Los Rios P., Jelezarov I., Trappe V. (2014). Hydrophobic hydration of poly-N-isopropyl acrylamide:
A matter of the mean energetic state of water *Sci*. Rep..

[28] Summa C. M., Langford D. P., Dinshaw S. H., Webb J., Rick S. W. (2024). Calculations of Absolute Free Energies,
Enthalpies, and Entropies for Drug Binding. J. Chem.Theory Comput..

[29] Yadav R., Paul B. K., Mukherjee S. (2023). Sequestration
of Drugs from Biomolecular and Biomimicking Environments: Spectroscopic
and Calorimetric Studies. Colloids and Interfaces..

[30] Moulik S. P., Naskar B., Rakshit A. K. (2019). Current
status of enthalpy-entropy compensation phenomenon. Curr. Sci..

[31] Cornish-Bowden A. (2002). Enthalpy–entropy
compensation:
a phantom phenomenon. J. Biosci..

[32] Zhao X. F., Wang J., Liu G. X., Fan T. P., Zhang Y. J., Yu J., Wang S. X., Li Z. J., Zhang Y. Y., Zheng X. H. (2015). Binding
mechanism of nine N-phenylpiperazine derivatives and α1A-adrenoceptor
using site directed molecular docking and high performance affinity
chromatography. RSC Adv..

[33] Schneider, H. J. , Yatsimirsky, A. Principles and Methods in Supramolecular Chemistry; Wiley: New York, 2000.

[34] Sarkar A., Kellogg G. E. (2010). Hydrophobicity-shake flasks, protein
folding and drug
discovery. Current topics in medicinal chemistry.

[35] Rupreo V., Tissopi R., Baruah K., Roy A. S., Bhattacharyya J. (2024). Multispectroscopic and Theoretical
Investigation on the Binding Interaction of a Neurodegenerative Drug,
Lobeline with Human Serum Albumin: Perturbation in Protein Conformation
and Hydrophobic–Hydrophilic Surface. Molecular Pharmaceutics..

[36] Roskoski R. (2021). Hydrophobic and polar interactions
of FDA-approved small molecule protein kinase inhibitors with their
target enzymes Pharmacol. Res..

[37] Baldwin R.
L. (2007). Energetics of Protein
Folding. J. Mol. Biol..

[38] Falanga A., Bellavita R., Braccia S., Galdiero S. (2024). Hydrophobicity: The
door to drug delivery. J. Pept Sci..

[39] Shaker D. S., Ishak R. A. H., Ghoneim A., Elhuoni M. A. (2019). Nanoemulsion: A
Review on Mechanisms for the Transdermal Delivery of Hydrophobic and
Hydrophilic Drugs. Sci. Pharm..

[40] McGuckin M. B., Wang J., Ghanma R., Qin N., Palma S. D., Donnelly R. F., Paredes A. J. (2022). Nanocrystals as
a master key to deliver
hydrophobic drugs via multiple administration routes. J. Controlled Release.

[41] Summa C. M., Langford D. P., Dinshaw S.-H., Webb J., Rick S. W. (2024). Calculations
of Absolute Free Energies, Enthalpies, and Entropies for Drug Binding. J. Chem. Computation.

[42] Shimizu S., Chan H. S. (2002). Anti-cooperativity and cooperativity
in hydrophobic
interactions: Three-body free energy landscapes and comparison with
implicit-solvent potential functions for proteins. Proteins.

[43] Xian W., Connolly P. J., Oslin M., Hausrath A. C., Osterhout J. J. (2006). Fundamental
processes of protein folding: Measuring the energetic balance between
helix formation and hydrophobic interactions. Protein Sci..

[44] Gerstman B. S., Chapagain P. P. (2005). Self-organization in protein folding and the hydrophobic
interaction. J. Chem. Phys..

[45] MacCallum J. L., Tieleman D. P. (2011). Hydrophobicity scales:
a thermodynamic looking glass
into lipid–protein interactions. J. Trends
Biochem. Sci..

[46] Lomize A. L., Pogozheva I. D., Lomize M. A. (2007). The role of hydrophobic
interactions in positioning of peripheral proteins in membranes. BMC Struct Biol..

[47] Roth C. M., Neal B. L., Lenhoff A. M. (1996). Van der Waals Interactions Involving
Proteins. Biophys. J..

[48] Malham R., Johnstone S., Bingham R. J., Barratt E., Phillips S. E., Laughton C. A., Homans S. W. (2005). Strong solute–solute dispersive
interactions in a protein–ligand complex. J. Am. Chem. Soc..

[49] Sung S.-S. (2015). Peptide
folding driven by Van der Waals interactions. Protein Sci..

[50] Zhdanov V.
P. (2019). Nanoparticles
without and with protein corona: van der Waals and hydration interaction. J. Biol. Phys..

[51] Roth C. M., Neal B. L., Lenhoff A. M. (1996). Van der
Waals interactions involving proteins. Biophys.
J..

[52] Roth C. M., Neal B. L., Lenhoff A. M. (1996). Van der
Waals interactions involving
proteins. Biophys. J..

[53] Dickinson E. (2016). Exploring
the frontiers of colloidal behaviour where polymers and particles
meet. Food Hydrocolloids.

[54] Galano-Frutos J. J., Sancho J. (2024). Energy, water, and
protein folding: A molecular dynamics-based
quantitative inventory of molecular interactions and forces that make
proteins stable. Protein Sci..

[55] Baldwin R. L., Rose G. D. (2016). How the Hydrophobic
Factor Drives Protein Folding. Proc. Natl. Acad.
Sci. U. S. A..

[56] Malham R., Johnstone S., Bingham R. J., Barratt E., Phillips S. E., Laughton C. A., Homans S. W. (2005). Strong solute–solute dispersive
interactions in a protein–ligand complex. J. Am. Chem. Soc..

[57] Newberry R. W., Raines R. T. (2019). Secondary Forces in Protein Folding *ACS Chem*. Biol..

[58] MacCallum J. L., Tieleman D. P. (2011). Hydrophobicity scales: a thermodynamic
looking glass
into lipid–protein interactions. Trends
Biochem. Sci..

[59] Wolfenden R. (2007). Experimental
measures of amino acid hydrophobicity and the topology of transmembrane
and globular proteins. J. Gen. Physiol..

[60] Radzicka A., Wolfenden R. (1988). Comparing
the polarities of the amino-acids - side-chain
distribution coefficients between the vapor-phase, cyclohexane, 1-octanol,
and neutral aqueous-solution. Biochem..

[61] Wimley W. C., White S. H. (1996). Experimentally determined
hydrophobicity scale for
proteins at membrane interfaces *Nat*. Struct. Biol..

[62] Wimley W. C. (1996). Solvation energies of amino acid side chains and backbone in a family
of host-guest pentapeptides. Biochem..

[63] MacCallum J. L., Tieleman D. P. (2011). Hydrophobicity scales:
a thermodynamic looking glass into lipid–protein interactions. Trends Biochem. Sci..

[64] Alhankawi A. R., Al-Husseini J. K., Spindler A., Baker C., Shoniwa T. T., Ahmed M., Chiarelli P. A., Johal M. S. (2022). The Relationship
between Hydrophobicity and Drug-Protein Binding in Human Serum Albumin:
A Quartz Crystal icrobalanceStudy. Biophysica.

[65] Al-Husseini J. K., Stanton N. J., Selassie C. R., Johal M. S. (2019). The binding of drug
molecules to serum albumin: the effect of drug hydrophobicity on binding
strength and protein desolvation. Langmuir.

[66] Schneider H.-J. (2015). Dispersive
interactions in solution complexes. Acc. Chem.
Res..

[67] Biedermann F., Nau W. M., Schneider H.-J. (2014). The Hydrophobic Effect Revisited—Studies
with Supramolecular Complexes Imply High-Energy Water as a Noncovalent
Driving Force. Angew. Chem., Int. Ed..

[68] Assaf K. I., Nau M. (2023). Dispersion Interactions
in Condensed Phases and inside Molecular
Containers. Acc. Chem. Res..

[69] Baron R., Setny P., McCammon J. A. (2010). How Can
Hydrophobic Association Be
Enthalpy Driven?. J. Am. Chem. Soc..

[70] Fenley A. T., Henriksen N. M., Muddana H. S., Gilson M. K. (2014). Bridging calorimetry
and simulation through precise calculations of cucurbituril–guest
binding enthalpies. J. chemical theory computation.

[71] Paul B. K. (2022). Classical
vs. nonclassical hydrophobic interactions underlying various interaction
processes. Aplication of isothermal titration calorimetry. ChemicalPhysicsImpact.

[72] Syme R., Dennis C., Phillips S. E. V., Homans S. W. (2007). Origin of Heat Capacity
Changes in a “Nonclassical”Hydrophobic Interaction. ChemBioChem..

[73] Sun Q. (2022). The Hydrophobic
Effects: Our Current Understanding. Molecules.

[74] Rego N. B., Patel A. J. (2022). Understanding Hydrophobic
Effects: Insights from Water
Density Fluctuations *Ann*. Rev.
Cond. Matt.Phys..

[75] DeLorbe J. E., Clements J. H., Teresk M. G., Benfield A. P., Plake H. R., Millspaugh L. E., Martin S. F. (2009). Thermodynamic and structural effects
of conformational constraints in protein–ligand interactions.
Entropic paradoxy associated with ligand preorganization. J. Am. Chem.Soc. Society.

[76] Usenik A., Leko K., Peroković V. P., Car Ž., Ribić R., Pičuljan K., Požar J. (2023). Hydrophobically driven hosting–What
about the guest?. J. Mol. Liq..

[77] Biedermann F., Nau W. M., Schneider H.-J. (2014). The Hydrophobic Effect Revisited—Studies
with Supramolecular Complexes Imply High-Energy Water as a Noncovalent
Driving Force. Angew. Chem., Int. Ed..

[78] Metherell A. J., Cullen W., Williams N. H., Ward M. D. (2018). Binding of Hydrophobic
Guests in a Coordination Cage Cavity is Driven by Liberation of “High-Energy”
Water. Chem.—Eur. J..

[79] Caldeweyher E., Ehlert S., Hansen A., Neugebauer H., Spicher S., Bannwarth C., Grimme S. (2019). A Generally Applicable
Atomic-Charge Dependent London Dispersion Correction. J. Chem. Phys..

[80] Verbaro D., Ghosh I., Nau W. M., Schweitzer-Stenner R. (2010). Discrepancies
between conformational distributions of a polyalanine peptide in solution
obtained from molecular dynamics force fields and amide I′
band profiles. J. Phys. Chem. B.

[81] Tóth G., Murphy R. F., Lovas S. (1999). Simulated
annealing studies on aromatic-amide
interaction in Phe-Gly-Gly tripeptide using different force fields. Internet J. Chem..

[82] Lii J.-H., Allinger N. L. (1991). The MM3 force field
for amides, polypeptides and proteins. J. Comput.
Chem..

[83] Chapman D. E., Steck J. K., Nerenberg P. S. (2014). Optimizing protein–protein
van der Waals interactions for the AMBER ff9x/ff12 force field. J. Chem. Theory Comput..

[84] Goldfarb N. E., Ohanessian M., Biswas S., McGee T. D., Mahon B. P., Ostrov D. A., Dunn B. M. (2015). Defective
hydrophobic sliding mechanism and active site expansion in HIV-1 protease
drug resistant variant Gly48Thr/Leu89Met: mechanisms for the loss
of saquinavir binding potency. Biochemistry.

[85] Malham R., Johnstone S., Bingham R. J., Barratt E., Phillips S. E. V., Laughton C. A., Homans S. W. (2005). Strong Solute-Solute Dispersive Interactions
in a Protein-Ligand Complex. J. Am. Chem. Soc..

[86] Ali M. S., Al-Lohedan H. A. (2023). Interactions
of lysozyme with hydrophobic and hydrophilic
non-steroidal anti-inflammatory drugs: Spectroscopic and molecular
docking analyses. J. Molecular Liquids A.

[87] Salonen L. M., Ellermann M., Diederich F. (2011). Aromatic rings in chemical and biological
recognition, energetics and structures. Angew.
Chem., Int. Ed..

[88] López J. J., García-Colunga J., Pérez E. G., Fierro A. (2018). Methylpiperidinium iodides as novel antagonists for
α7 nicotinic acetylcholine receptors *Front*. Pharmacol..

[89] Osterhout J. J. (2005). Understanding
protein folding through peptide models. Protein
Pept. Lett..

[90] Caporale A., Adorinni S., Lamba D., Saviano M. (2021). Peptide-Protein Interactions:
From Drug Design to Supramolecular Biomaterials. Molecules..

[91] Tatko C. D., Waters M. L. (2004). Effect of halogenation
on edge-face aromatic interactions
in a β-hairpin peptide: Enhanced affinity with lodo-substituents *Org*. Lett..

[92] Hasselgren C., Oprea T. I. (2024). Artificial Intelligence
for Drug Discovery: Are We
There Yet?. Annu. Rev. Pharmacol. Toxicol..

[93] Blanco-Gonzalez A., Cabezon A., Seco-Gonzalez A., Conde-Torres D., Antelo-Riveiro P., Pineiro A., Garcia-Fandino R. (2023). The role of
AI in drug discovery: challenges, opportunities, and strategies. Pharmaceuticals.

[94] Sarkar C., Das B., Rawat V. S., Wahlang J. B., Nongpiur A., Tiewsoh I. (2023). Artificial
intelligence and machine learning technology driven modern drug discovery
and development *International*. J. Molecular Sci..

[95] Backenköhler M., Groß J., Wolf V., Volkamer A. (2024). Guided docking as a
data generation approach facilitates structure-based machine learning
on kinases. J: Chem. Information Modeling.

