# Supplementary material for: Problematic Attributions of Entropic and Hydrophobic Effects in Drug Interactions
Source: ACS Bio Med Chem Au. 2025 Apr 11;5(3):334–41. doi: 10.1021/acsbiomedchemau.4c00148 (PMC12183581; doi:10.1021/acsbiomedchemau.4c00148)
Supplement: Supplementary file 1 [file bg4c00148_si_001.pdf]

Supplemental Material to
Hans-Jörg Schneider
Problematic Attributions of Entropic and Hydrophobic Effects in Drug Interactions

Figure S1. Ion pair association free energies in water : average 5 kJ/mol per salt bridge

Figure S2. Ion pair stability in water as function of ionic strength . Debye-Hückel-correlation

Figure S3: Ion pair distance dependence of binding free energy ΔG , measured in water

Figure S4. Solvent effects on ion pairing /salt bridges . Linear dependence of the association energy ΔG (kJ/mol) of Et_4NBr on dielectric constant with 1/e

Figure S5. Hydrogen bond □□G increments reflect electrostatic interactions: correlation between hydrogen bond increments ED and free energies ΔG of crown and cryptand complexes

F

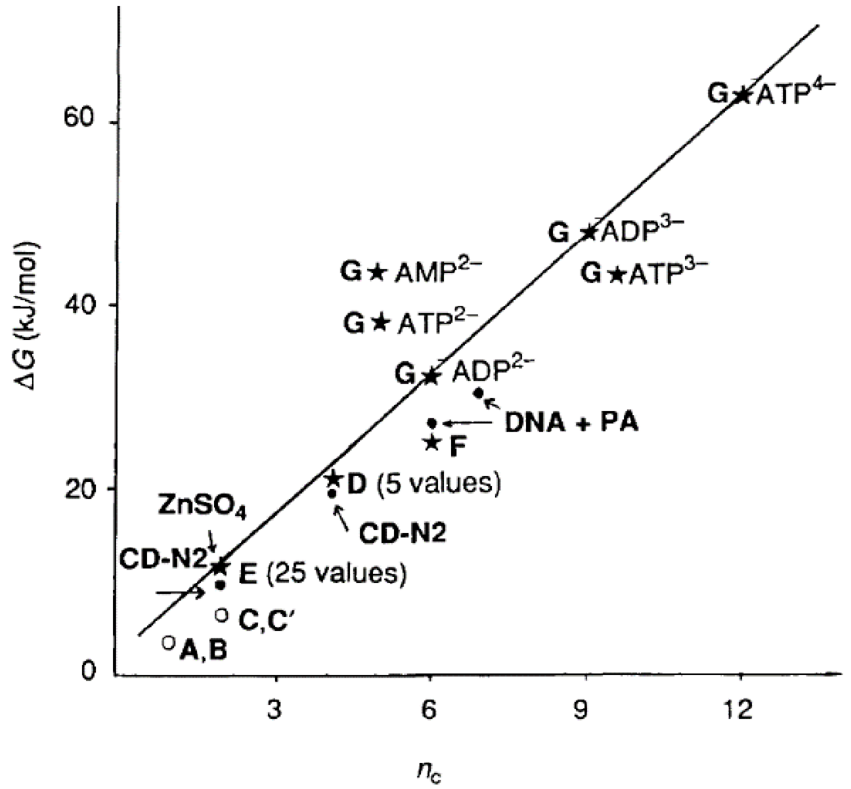

Figure S1 Ion pair association free energies in water : **average 5 kJ/mol per salt bridge**

ΔG [kJ/mol] *vs*. number n of salt bridges in ion pairs, in water at average ion strength ***I*** (dependence on ***I*** see Figure **S2**). A, B and C,C0, complexes of a tetraphenolate cyclophane (4 ˇ) with Me_4N^+ and an azoniacyclophane with mono- and dianionic naphthalene derivatives; D, anionic (sulfonate or carboxylate) with cationic (ammonium) triphenylmethane derivatives; E, organic dianions with organic dications; F, cationic azamacrocyle (6 á charges) with aliphatic dicarboxylates; G, cationic azacrowns with adenosinemono-,di- and triphosphates. Adapted with permission from ref (16). Copyright (2016), ACS.

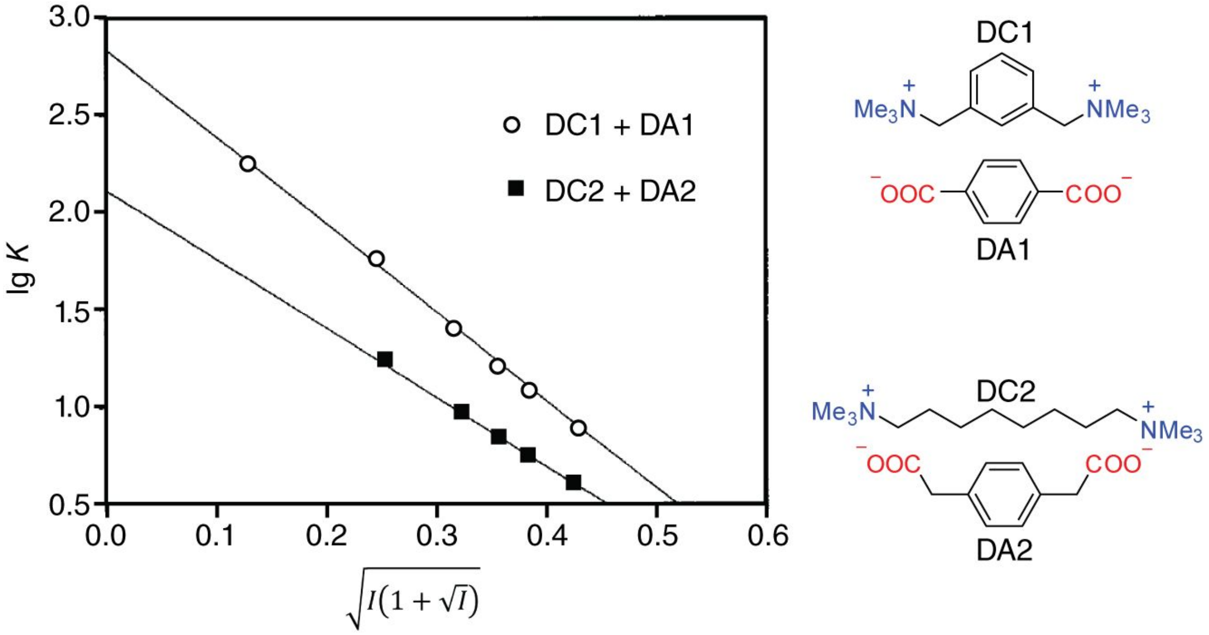

Figure 2- Ion pair stability in water as function of ionic strength . Debye-Hückel-correlation of association constants (as lg *K*) of ion pairs CP and CP2 and the ionic strength measure $\sqrt{I(1+\sqrt{I})}$ of the aqueous medium. Reprinted with permission from ref. Hossain, M. A.; Schneider, H.-J. Flexibility, association constants, and salt effects in organic ion pairs: How single bonds affect molecular recognition. *Chemistry* **1999**, *5*, 1284-1290 For detailed analysis of DG dependence of alkali and earth alkali salts see Sammartano, S. et al Thermodynamic parameters for the binding of inorganic and organic anions by biogenic polyammonium cations. *Talanta* **2001**, *54*, 1135-1152,;. Weak alkali and alkaline earth metal complexes of low molecular weight ligands in aqueous solution. Reproduced with permission from ref (16). Copyright (2016), ACS.

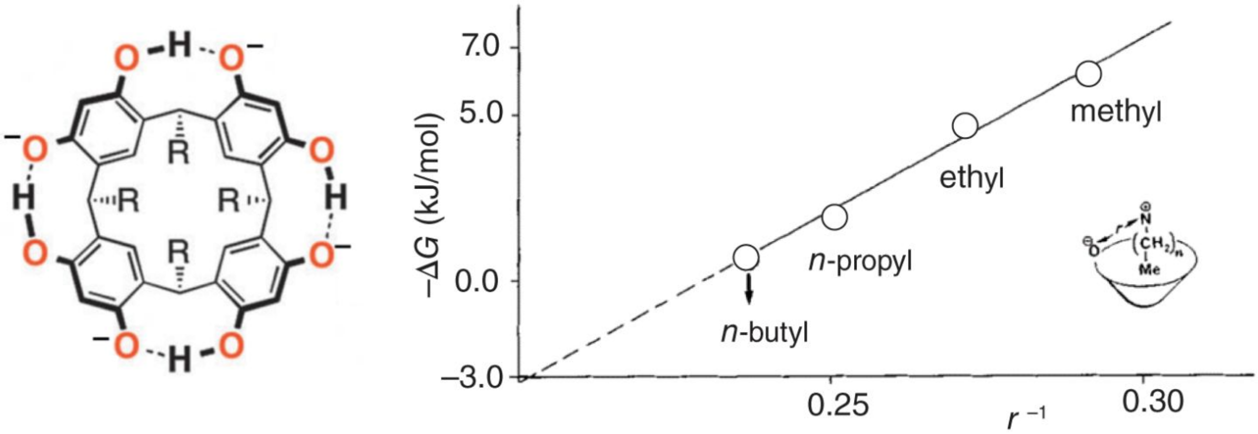

Figure S3: **Ion pair distance dependence of binding free energy Δ*G*** measured in water with complexes of a tetraphenolate host and tetraalkylammonium ions R_4N^+ (R = Me, Et, *n*-propyl, *n*-butyl). Reprinted with permission from ref. Copyright 1988 with permission American Chemical Society. Schneider, H. J.; Guettes, D.; Schneider, U. Host-guest complexes with water-soluble macrocyclic polyphenolates including induced fit and simple elements of a proton pump. Adapted Reproduced with permission from ref (16). Copyright (2016), ACS.

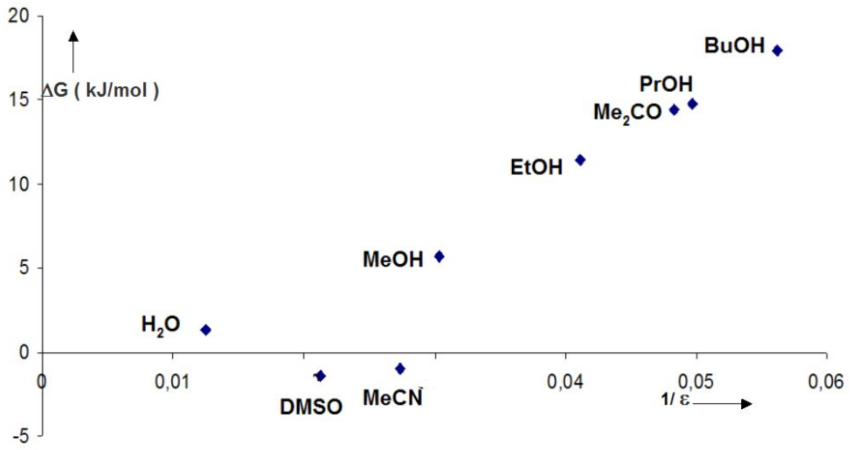

Figure S4 Solvent effects on ion pair. Linear dependence of the association energy Δ*G* (kJ/mol) of Et_4NBr on dielectric constant with 1/ □□ for protic media. Solvents such as DMSO are known for an exceptional strong cation coordination. Reprinted with permission from ref. [14]. Copyright 2009 Wiley VCH. Schneider, H.-J. Binding Mechanisms in Supramolecular Complexes. Adapted with permission from *Angew. Chem. Int. Ed.* **2009**, *48*, 3924-3977. Coypight woo9 Wiley VCH.

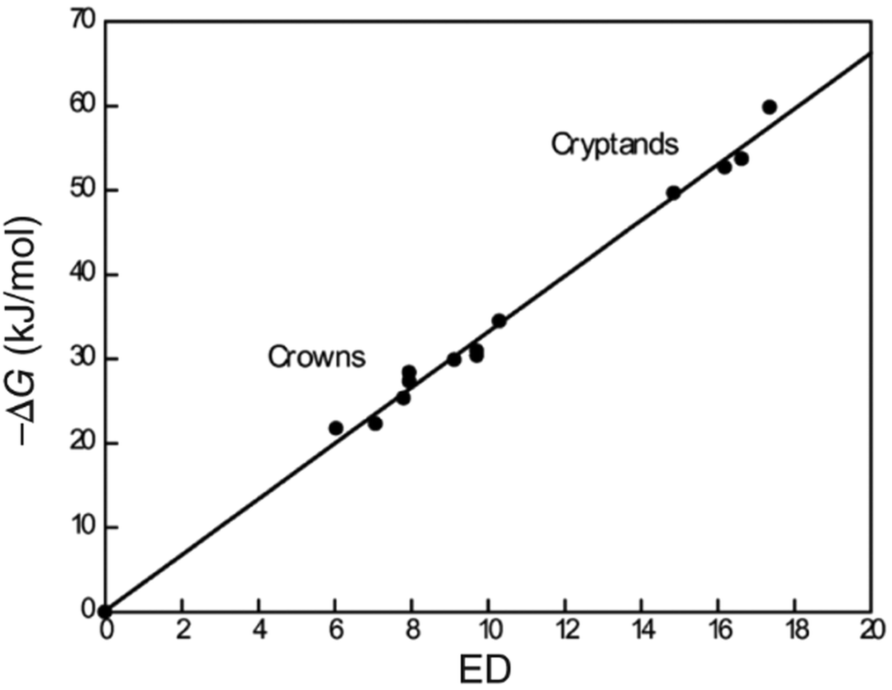

Figure S5 Hydrogen bond ΔΔG increments reflect electrostatic interactions: correlation between such H-bond increments ED and free energies ΔG of crown and cryptand ether potassium (K^+) complexes in methanol. Reproduced with permission from ref (16). Copyright (2016), ACS.

Table S1. [a] Typical Binding Free Energies for Important Noncovalent Interactions from supramolecular complexes

interaction type	system	$-\Delta G$[kJ/mol]
**salt bridges and ion pairs**	inorganic salts in H_2O	5 to 6
	organic ions in H_2O	5 to 8
	ionic groups at protein surface	0 to 7
	ionic groups buried in protein core	12 to 20
**hydrogen bonds**	PhO-H···amide in CCl_4	11
neutral	PhO-H···amide in $CDCl_3$	6 to 8
	amide–amide in $CDCl_3$	5 to 8
	amide–amide in protein core	2 to 10
	C-H···indole in $CDCl_3$	4
	p-F-PhO-H···F-CHR_2 in CCl_4	6
	p-F-PhO-H···I-CHR_2 in CCl_4	4
	Thr···Ser in protein core	3
with cations	$^+$N-H···O-CH_2 (18-crown-6) in H_2O	3
	+N-H···O-CH_2 (18-crown-6) in MeOH	8
with anions	ureas···Cl^- in DMSO	9
	ureas···COO^- in DMSO	20
	pyrroloamides···Cl^- in DMSO	13
	pyrroloamides···COO^- in DMSO	21
	squaramides···F^- in CH_3CN	46
	squaramides···Cl^- in CH_3CN	35
	squaramide-cleft···Cl^- in $CHCl_3$ (per squaramide)	31 to 39
	C_6H_5O–H···$^-OC_6H_4$-*p*-NO_2 in H_2O	17
	Thr···Asp in protein core	7
**halogen bonds**	C_6F_5I···$Cl^- > Br^- > I^-$ in acetone	7 to 9
	$C_8F_{17}I$···$Cl^- > Br^- > I^-$ in acetone	14 to 19
	Ph-F···O=C-NR_2*orthogonal* in C_6D_6	1
	Ph–Cl···O=C-NR_2 in protein pocket	6
	Ph-I···O=C-NR_2 in protein pocket	10
**electrostatic**	e-rich clip···$C_6H_2(CN)_4$ in $CDCl_3$/acetone	20
	pillar[5]arene···NC–CH_2CH_2–CN in *o*-xylene	34
	pillar[5]arene···NC–CH_2CH_2-CN in CH_3CN	14
	“blue-box”···Phe in H_2O	9

**interaction type**	**system**	**$-\Delta G$[kJ/mol]**
**dispersive and stacking**	porphyrin···pyridine in H_2O	7
	porphyrin···quinoline in H_2O	17
	H_2O···Kr in gas phase	2.0
	cucurbit[5]uril···Xe in H_2O	26[a]
	cryptophane···Xe in H_2O	30[a]
	cucurbit[6]uril···CH_2 – increment, in H_2O	~6[a]
**cation−π**	indole···pyridinium in H_2O	2
	phenyl···alkylammonium in H_2O	1 to 3
	cyclophanes···quinolinium in H_2O	2 to 3 per contact
	various association complexes in H_2O	1.5 per contact
**anion−π**	RC_6F_5···Cl^- in acetone	7
	$3,5(NO_2)C_6H_4$-···Cl^- in C_6D_6	3 to 4
	$3,5(NO_2)C_6H_4$-···Cl^- in MeCN	2 to 3
	phenyl···RSO_3^- in H_2O	1 to 2
**hydrophobic**	alkane···flat surface in water	1 to 3 per CH_2
	various host–guest complexes in water	~0 to 60

[a]

Corrected for $\Delta G_{solvation}$.

[a] Reproduced with permission from ref (16). Copyright (2016), ACS.

Binding increments for noncovalent interactions have to be treated with care, because (*i*) the system of interest should be as closely related as possible to reference systems for which the binding energies were determined, (*ii*) reported binding increments, e.g., that for a hydrogen-bond, may or may not be corrected for other, simultaneously occurring interactions and effects that were overlaying/masking the "pure" interaction type of interest.
